# Exploring transcutaneous electrostimulation for proprioceptive feedback in upper limb prostheses: a pilot study

**DOI:** 10.1186/s12984-025-01772-z

**Published:** 2025-11-17

**Authors:** Eleonora Fontana, Gabriele Natili, Manuel G. Catalano, Giorgio Grioli, Antonio Bicchi

**Affiliations:** 1 Centro di Ricerca E. Piaggio and Department of Information Engineering, University of Pisa, 56122 Pisa, Italy; 2 Soft Robotics for Human Cooperation and Rehabilitation, Istituto Italiano di Tecnologia, 16163 Genova, Italy

**Keywords:** Transcutaneous electrical stimulation, Proprioception, Haptics and haptic interfaces, Prosthetics, Exoskeletons

## Abstract

**Background:**

Myoelectric upper-limb prosthetic users frequently encounter challenges related to proprioceptive deficits, which can impair their ability to perceive limb position and movement. As a result, there has been growing interest in developing strategies to enhance sensory feedback in these users. One promising approach is the use of transcutaneous electrostimulation (TES) to modulate kinesthetic perception and improve proprioception.

**Methods:**

This study consists of two experiments. In the first experiment, TES was applied to the finger flexor muscles of ten able-bodied participants and one prosthetic user to explore its effects on kinesthetic perception. In the second experiment, TES was combined with a myoelectric prosthesis in an object discrimination task. Three able-bodied participants and one prosthetic user were asked to distinguish between two cylinders of different sizes using their prosthetic limb, with the goal of assessing the potential of TES to enhance feedback during functional tasks.

**Results:**

In the first experiment, TES evoked the illusion of finger extension, exceeding 1 cm in able-bodied participants and reaching up to 4 mm in the prosthetic user. In the second experiment, TES facilitated object discrimination, with able-bodied participants achieving over 80% accuracy and the prosthetic user exceeding 85% under different conditions.

**Conclusion:**

TES holds promise as a non-invasive approach for enhancing proprioceptive feedback in prosthetic users, with initial results indicating its potential to improve kinesthetic perception. However, challenges such as the occurrence of unintended tactile sensations highlight the need for further refinement of the technique. Future studies will be needed to refine TES parameters and to investigate its integration within more structured experimental settings, in order to better evaluate its potential in real-world prosthetic applications.

## Background

Humans perform various tasks daily, such as grasping and manipulating objects, interpreting tactile information, and interacting with the environment. Losing an upper limb, whether due to trauma, pathology, or congenital conditions, significantly impacts an individual’s quality of life.

Prosthetic devices have been developed to help compensate for such conditions, partially restoring lost motor functions. However, they cannot yet fully replicate the sensory perceptions of the missing limb. The absence of sensory feedback in prosthetic users results in less intuitive control, the need for constant visual monitoring, increased cognitive load, lower acceptance of the limb, and less efficient control. This leads to higher energy consumption and shorter battery life. Notably, due to the lack of sensory feedback, approximately a quarter of prosthetic technologies are rejected by users, with a 23% rejection rate in adults for myoelectric upper-limb prostheses [1]. Consequently, research efforts have increasingly focused on the restoration of sensory feedback. Most studies emphasize recovering tactile sensations [2, 3], while the artificial generation of other modalities, such as proprioceptive feedback and cutaneous stimuli, has been studied to a much lesser extent. Proprioception, or kinesthesia, is particularly crucial as it allows individuals to perceive the position and movement of their body parts in space without visual input. Deficits in proprioception can severely impair motor control, as demonstrated in various human and animal studies [4, 5]. Moreover, the absence of proprioception can lead to errors in direction and amplitude during reaching tasks and delays in muscular strategies for postural control [6, 7]. The lack of explicit proprioceptive feedback degrades system functionality, resulting in non-intuitive control. However, providing proprioceptive feedback, even through sensory substitution methods [8], has the potential to enhance control and overall system performance.

**Fig. 1:**
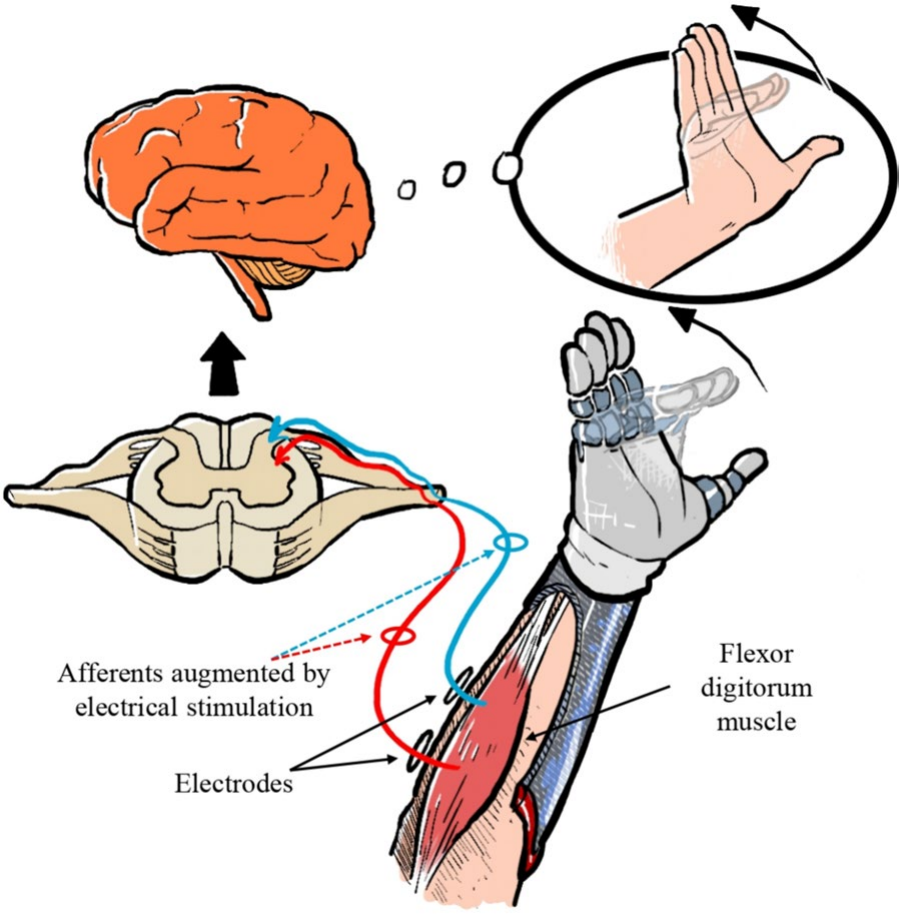
Schematic representation of the objective of the study: to explore the use of transcutaneous electrostimulation to enhance proprioceptive feedback in trans-radial prosthetic users. The illustration depicts how electrical stimulation is applied to the residual flexor muscle of the forearm. This stimulation aims to evoke sensory feedback that generates the perception of finger movement in the robotic hand, potentially improving the user’s awareness of hand position and motion

Although proprioception involves the interaction of multiple sensory modalities, the primary kinesthetic receptors include muscle spindles and Golgi tendon organs [9–11]. Various neuromodulation techniques have been explored to generate or modulate kinesthetic perception by altering the firing rate of proprioceptive afferents. One common invasive approach involves implanting a pulse generator directly at peripheral nerves [12, 13]. Non-invasive techniques have also gained attention, particularly the use of vibrotactile actuators, which have been extensively studied for their effectiveness in conveying tactile information without requiring muscle activation [8, 9, 14, 15].

Less common non-invasive methods include skin stretching [16, 17], enhanced tactile feedback [2, 18], and sensory substitution [19]. Vibrotactile feedback provides intuitive sensations, which can improve the user’s experience. Recent studies have demonstrated that vibrotactile feedback can effectively enhance movement perception in individuals with upper limb amputations, potentially improving motor control and functional performance in prosthetic users [15].

In 2021, Park et al. [20] proposed an innovative non-invasive technique utilizing transcutaneous electrostimulation (TES) to address proprioceptive deficits associated with neurotraumas and neurodegenerative diseases. This approach involves placing electrodes on the elbow flexor muscles to deliver electrical stimulation that targets specific physiological afferent pathways. By modulating the activity of these muscles, the technique aims to create the illusion of elbow joint extension, thereby enhancing proprioceptive feedback. Compared to mechanical vibration, electrostimulation offers distinct advantages for providing sensory feedback in prosthetic devices. Despite the potential discomfort associated with the use of electrodes or stimulation devices, electrostimulation excels in offering a smaller form factor, a robust contact interface, and high controllability. These features enhance its suitability for wearable devices. Moreover, electrostimulation provides benefits in terms of latency, consistency, and ease of implementation, making it a promising candidate for proprioceptive restoration in individuals with limb loss. However, to date, the technique presented in [20] has only been utilized to induce the illusion of elbow joint extension, thus limiting its applicability to a specific case.

Recent advances have extended TES-based proprioceptive feedback beyond the elbow. For instance, Han et al. [21] delivered proprioceptive information for wrist flexion–extension using a multichannel array of surface electrodes and a spatial coding strategy, whereby different joint positions were mapped to different electrode sites. Although this approach yielded high discrimination accuracy, it primarily relied on cutaneous stimulation at a superficial level, without directly eliciting a muscle-length illusion. More recently, Ravichandran et al. [22] proposed an electrotactile feedback paradigm for finger aperture, using frequency-modulated cutaneous stimulation at the fingertips to improve motor accuracy and training retention. In contrast to these methods, our study specifically targets muscle afferents in the forearm to evoke proprioceptive illusions of finger flexion, thereby exploring whether such muscle-based TES feedback can be integrated into prosthetic hand control. Our contribution lies in investigating the utility of TES-induced proprioceptive feedback for encoding prosthetic hand aperture in a pilot setting, as depicted in Fig. 1. The primary aim of this study is to provide a preliminary investigation into the feasibility of delivering proprioceptive feedback via TES applied to the finger flexor muscles. Due to the novelty of the approach, and the need for initial technical validation, we designed the protocol as a pilot study, focusing on early-stage perceptual effects in a controlled environment.

The study is organized into two main experiments. In the first experiment, we validate the efficacy of TES on the finger flexor muscles by adapting and refining the protocol established by Park et al., which was originally designed for elbow flexion. Participants were asked to replicate the pose of their right hand with their left hand (the prosthetic hand for the prosthesis user) while TES was applied to the right hand. The results indicate that TES effectively induces aliasing in proprioceptive perceptions of hand position during the task.

In the second experiment, we explore the use of TES in combination with a myoelectric prosthesis to provide proprioceptive feedback during cylinder discrimination tasks. The accuracy achieved in discriminating between the two cylinders indicates that TES-based proprioceptive cues can be perceived and interpreted by users under controlled conditions. These results support the feasibility of this approach and motivate further investigation in more functionally representative settings.

Overall, these findings indicate that TES when applied to the finger flexor muscles has significant potential for improving proprioceptive feedback in myoelectric prostheses. However, the main limitations include the use of only one prosthetic user, who exhibited limited response to stimulation and lacked a true baseline perception of the missing limb. Anatomical differences due to congenital aplasia and the influence of tactile sensations further impacted accuracy, emphasizing the need for optimization. Further research is needed to refine this technique and explore its applicability in more complex tasks and with a broader range of prosthetic users.

## Methods

### TES affects hand proprioception

The primary objective of this first part was to evaluate the illusory effect of TES on the perception of finger movements. By closely adhering to Park et al.’s experimental procedure [20], particularly in terms of the duration of the stimulations and data processing, and with adjustments to focus on hand-matching tasks rather than arm-matching, we aimed to assess the potential for proprioceptive modulation in finger movements. Importantly, the inclusion of a prosthetic user allowed us to evaluate its application in prosthesis control, thus exploring the effectiveness of the TES in both scenarios.

#### Participants

This study involved a sample of ten able-bodied participants (mean age: 26, range: 25-27 years, gender: 2 females, 8 males) to validate the feasibility and effectiveness of the protocol for hand joint extension. The participants did not have any neuromuscular disorders and had no history of hand or arm injuries. Following the initial testing with able-bodied participants, the study included an individual with congenital amputation (age: 43 years old, gender: female). The amputee participant was born with *aplasia*, which prevented the development of her left forearm. All the experimental procedures were approved by the Committee on Bioethics of the University of Pisa (Review N. 53/2023) for testing a single prosthetic participant under a limited exploratory protocol. All participants gave their written informed consent prior to participation. The study was conducted in accordance with the principles outlined in the Declaration of Helsinki.

#### Experimental setup

**Fig. 2:**
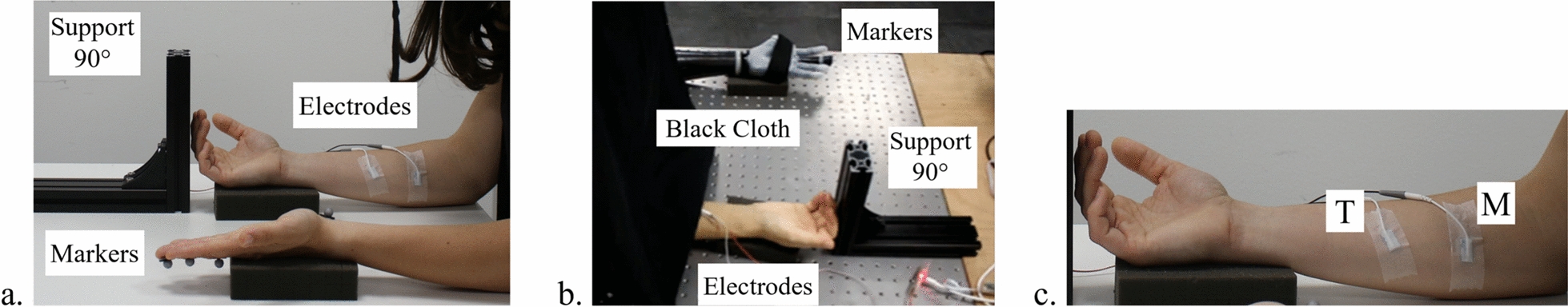
Experimental setup for assessing participants’ ability to match hand positions and movements during TES, showing (**a**) an able-bodied participant and (**b**) a prosthetic user with electrodes attached to the right arm, the hand secured in a semi-closed, supine posture using external support; and (**c**) electrode placement on the muscle belly (M) and distal myotendinous junction (T) of the right arm. Vicon markers are placed to track movement during the task

For this experimental study, we utilized an integrated setup consisting of a PC, a power supply unit (EA-PS 2042-10 B), and a controller programmed to generate biphasic square-wave output with a 50% duty cycle, allowing for operator-regulated amplitude and frequency. The setup also included a pair of reusable self-adhesive gel surface electrodes (TensCare, Axion - Leonberg, Germany), a digital oscilloscope (RTB2004, Rohde & Schwarz - Munich, Germany), and a marker-based motion capture system (Vicon Motion Systems - Oxford, UK) for recording movement. During the experiments, the participant sat at a table with both arms resting on its surface, and palms in a supine position. Electrodes were attached to the right arm, and the hand was secured in a specific resting posture using an external support. The left arm also rested on the table with the palm facing upward. Vicon markers were initially placed on both hands to record the position of each limb, after which they were removed from the right hand to proceed with the experiment. This setup is illustrated in Fig. 2a. Regarding the prosthetic user, stimulation was applied to the fully developed finger flexor muscle in the right arm, while hand-matching was performed using the prosthesis. TES was applied to the healthy forearm of the prosthetic user, rather than the residual limb, to isolate the effects of TES on kinesthetic perception without the added complexity of prosthetic-related factors. This approach allowed us to establish a baseline response to TES, providing a more controlled evaluation before applying stimulation to the residual limb in subsequent experiments. To ensure that visual feedback did not interfere with the induced perceptual effect, a black cloth was placed in front of the participant’s right hand, preventing any view of the finger positions during the matching task, as shown in Fig. 2b.

#### Electrode placement

In our study, we focused on a single muscle, the flexor digitorum profundus muscle, identifying two possible positions for electrode placement: the muscle belly (M) and the distal myotendinous junction area (T). Consequently, three possible combinations for the electrode pair were derived: M-M, T-T, and M-T. This approach allowed us to repeat the entire protocol for each participant three times, once for each electrode combination. The electrodes were customized with a small footprint (1.2cm$\times$0.8cm) to ensure precise localization of electrical stimulation and to identify optimal electrode positions with maximum efficiency. Conductive gel was applied beneath the electrodes to enhance conduction, and additional adhesive tape was used to secure the electrodes, ensuring stable skin contact during movements. For visual reference regarding the positioning of the electrodes on the right arm, please refer to Fig. 2c.

#### Marker placement and hand posture analysis

**Fig. 3:**
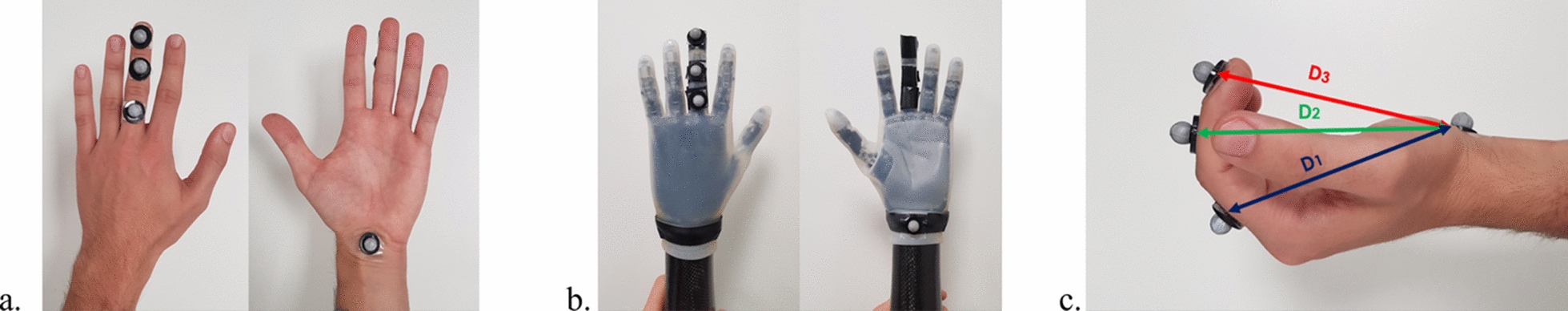
Schematic representation depicting (**a**) placement of Vicon markers on the hand and (**b**) on the prosthetic hand. The reference marker is positioned on the wrist, while the other three are arranged on the dorsum of the three phalanges of the middle finger. As shown in (**c**), these markers are utilized to calculate the parameters $\hbox {D}_1$, $\hbox {D}_2$, and $\hbox {D}_3$ which assess hand posture

In our experiments, we opted for a Vicon marker-based motion capture system to accurately track the position of the fingers in real-time. Thus, we defined a set consisting of four reflective markers (6.4 mm spherical markers): one reference marker placed at the wrist and three additional markers positioned on the dorsum of the three phalanges of the middle finger, as depicted in Fig. 3a. In order to assess hand posture, we calculated a single parameter, denoted as $\hbox {D}_{tot}$. This parameter is derived from the mean of three distances, $\hbox {D}_1$, $\hbox {D}_2$, and $\hbox {D}_3$, highlighted in Fig. 3c. Specifically, $\hbox {D}_3$ measures the distance from the fingertip to the reference marker, while $\hbox {D}_2$ and $\hbox {D}_1$ indicate the distances from the middle and proximal phalanges, respectively, to the same reference marker. It is important to note that all the linear measurements considered (i.e. $\hbox {D}_1$, $\hbox {D}_2$, and $\hbox {D}_3$) account for the joint angles of the phalanges. These distances have been explicitly determined using the law of cosines, thereby accounting for the specific angles assumed by the relevant joints. This ensures that our parameter $\hbox {D}_{tot}$ effectively reflects the hand posture, including the contributions of the finger joint angles.

Because TES induces a controlled contraction of specific muscle groups, leading to finger flexion or extension, Dtot provides a straightforward and continuous measure of the resulting biomechanical effect. As the finger flexes in response to stimulation, all three phalangeal markers move closer to the wrist, and Dtot decreases accordingly. Conversely, in the absence of stimulation or during finger extension, Dtot increases. Therefore, tracking changes in Dtot allows us to quantify the dynamic motor response elicited by TES with high temporal resolution.

The same marker positioning and calculation methodology were also applied to the prosthetic user, with the marker positioning details outlined in Fig. 3b.

#### Experimental procedure

Participants were instructed to relax their right arm and were blindfolded to eliminate visual bias. Electrodes and Vicon markers were placed according to the positions identified in previous sections.

1. **Calibration procedure for optimal stimulation parameters**
  To determine the optimal voltage and frequency settings for muscular stimulation, we conducted an initial calibration procedure. This procedure was specifically tailored to accommodate individual variations in stimulation tolerance and perception thresholds. Stimulation was applied to the finger flexor muscle for a standardized duration of 5 seconds. Initially, the voltage was gradually increased, starting from 5V at a fixed frequency of 100Hz, until participants reported the first perceptible electrotactile feedback, defining the perception threshold *Vth*. Subsequently, the voltage was further increased by one-volt increments until the participant experienced discomfort, establishing the maximum tolerable voltage *Vmax*. A similar approach was used to determine the optimal stimulation frequency. Five different frequencies were tested (30Hz, 100Hz, 300Hz, 1000Hz, and 3000Hz) while keeping the amplitude fixed at *Vmax*. It is important to note that standard psychophysical methods were not employed to measure sensory thresholds or to determine the stimulation amplitude, which represents a limitation of this study.
  Participants were requested to assess the induced proprioceptive illusion on a scale from 1 to 5 for each voltage and frequency. The scores were defined as follows: 1–nothing, 2–barely perceivable, 3–perceivable, 4–strong, 5–very strong. This subjective scale was used as an initial tool to quantify the perceived intensity of the effect evoked by TES. Although the scale did not explicitly distinguish between tactile and kinesthetic sensations, participants were encouraged to rate based on their personal interpretation of the effect, particularly focusing on the sense of finger movement. This approach allowed us to capture the participants’ perceptual experience in a simple and intuitive way. Based on these scores, for each of the three electrode positioning configurations (M-M, M-T, T-T), we identified the optimal voltage and frequency values for each participant. The optimal amplitude for each configuration was determined by selecting the amplitude corresponding to the highest perceived score; if multiple amplitudes resulted in the same score, the highest amplitude was chosen. Regarding the frequency, for each configuration, the value corresponding to the highest perceived score was selected. If multiple frequencies resulted in the same score, the frequency closest to 100 Hz was chosen, consistent with previous studies [23, 24].
2. **Hand-Matching task** As a preliminary step, markers were placed on both hands to record the resting position of the right hand, measuring $\hbox {D}_1$, $\hbox {D}_2$, and $\hbox {D}_3$, which were then used to obtain $\hbox {D}_{tot}$. After this step, the markers on the right hand were removed to prevent interference with the external supports used during the experiment. The Hand-Matching task was conducted at three predefined hand angles (45$^{\circ }$, 90$^{\circ }$, and 135$^{\circ }$), with the order of angles randomized across sessions. For each condition, the participant placed their right hand against an external support that constrained it to the designated angle, ensuring a stable reference position. The left hand, which remained free to move, was used to actively reproduce the perceived position of the right hand by matching its configuration. The task was conducted under three conditions: baseline (no stimulation), stimulation, and post-stimulation (aftereffect), in randomized order. A rest period of 5 seconds was included between flexions to reduce fatigue.
  During each condition, participants were asked to match the perceived posture of their right hand with their left hand while keeping visual feedback blocked. In the baseline phase, this matching was performed four times. During the stimulation phase, transcutaneous electrical stimulation (TES) was delivered, and the task was repeated twice. In the aftereffect phase, the matching was again performed twice, without stimulation.
  Throughout the task, the position of the left hand was continuously recorded. To quantify changes in hand positioning induced by TES, we computed the net change, defined as the difference between the D*tot* recorded during (or after) stimulation and the corresponding D*tot* obtained during the baseline measurement. To minimize potential adaptation effects, a 1-minute rest interval was maintained between sessions.
  This procedure is consistent with the general framework used in [20], although adapted to fit the specific goals and setup of the present study.

#### Data analysis

To evaluate the effects of TES on kinesthetic perception, we analyzed $\hbox {D}_{tot}$ values recorded from the participants’ left hand during the Hand-Matching task. The analysis followed a structured approach to ensure consistency and minimize artifacts in the data. First, we determined the effective duration of each measurement by identifying the start and end points of the hand-matching period. To reduce the impact of transient responses and movement undershoot, we excluded the first 1.5 seconds and the last 1 second of the recorded period. The remaining interval was used to compute the mean $\hbox {D}_{tot}$ value for each measurement. For each participant, we established a Baseline reference by averaging the first four hand-matching trials before stimulation. We then computed the mean $\hbox {D}_{tot}$ values separately for two key phases: the Stim-On, defined as the period during which TES was applied (averaging the two hand-matching trials with stimulation), and Stim-Off, defined as the post-stimulation period (averaging the last two hand-matching trials without stimulation). To determine whether stimulation significantly influenced hand-matching performance, we conducted statistical analyses. First, a normality test was performed to assess data distribution. We then conducted statistical tests to compare the Baseline vs. Stim-On and Baseline vs. Stim-Off conditions across all experimental sessions (i.e., $45^{\circ }$, $90^{\circ }$, and $135^{\circ }$ hand closure angles).

### TES displays prosthesis status

In the second experiment of our study, we aimed to explore the use of TES in combination with a myoelectric prosthesis to determine whether stimulating the neuromuscular spindles could generate a perception of the position of the robotic hand, even in the absence of visual input.

#### Participants

The participants comprised three able-bodied participants (mean age: 26, range: 25-27 years, gender: 2 females, 1 male), and one individual with congenital amputation who had also participated in the first experiment of the study. The choice to include only three able-bodied participants was driven by the primary aim of applying the validated technique to the prosthesis user. The three able-bodied participants were tested solely to validate the protocol before applying it to the prosthesis user. This preliminary validation on able-bodied participants was crucial to ensure the protocol efficacy before proceeding with the single available prosthesis user. Consequently, this small sample was not intended to provide significant statistical power, but rather to serve as a preliminary validation step. All the experimental procedures were approved by the Committee on Bioethics of the University of Pisa (Review N. 53/2023) for testing a single prosthetic participant under a limited exploratory protocol. All participants gave their written informed consent prior to participation. The study was conducted in accordance with the principles outlined in the Declaration of Helsinki.

#### Experimental setup

**Fig. 4:**
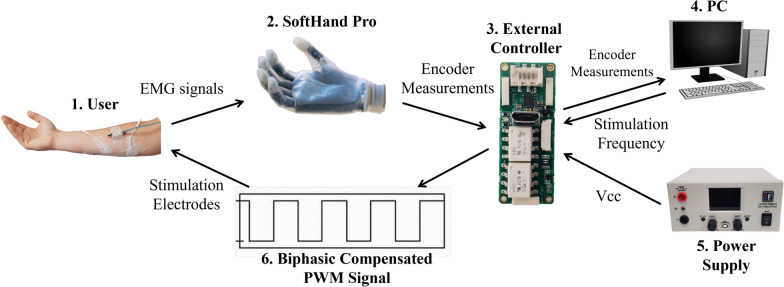
Schematic of the experimental setup. The participant wears two electrodes on their right arm, similar to the first part of the study. The SoftHand Pro prosthesis, equipped with an encoder, measures finger closure and sends the data to an external controller. The controller, powered by a Vcc supply, transmits measurements to a PC and receives the stimulation frequency, generating a biphasic PWM signal delivered to the user through the stimulation electrodes.

The experimental setup required for this section, as described below, is in addition to what is listed in Section 2.1.2. This setup includes the *SoftHand Pro* myoelectric prosthesis [25, 26] and two EMG electrodes. The participant wore the same two electrodes on their right arm as used in the first part of the study. Additionally, the prosthesis was equipped with an encoder that measured the degree of finger closure and sent this data to an external controller, powered by a Vcc power supply. The external controller sent the encoder measurements to the PC and received the corresponding stimulation frequency. The controller processed this input and generated a biphasic, compensated PWM signal with a 50% duty cycle and an amplitude equal to Vcc. This signal was then delivered to the user through the stimulation electrodes. For a comprehensive understanding of the overall process, please refer to Fig. 4, which provides a schematic representation of the control and communication flow between the different components.

#### Electrode placement

**Fig. 5:**
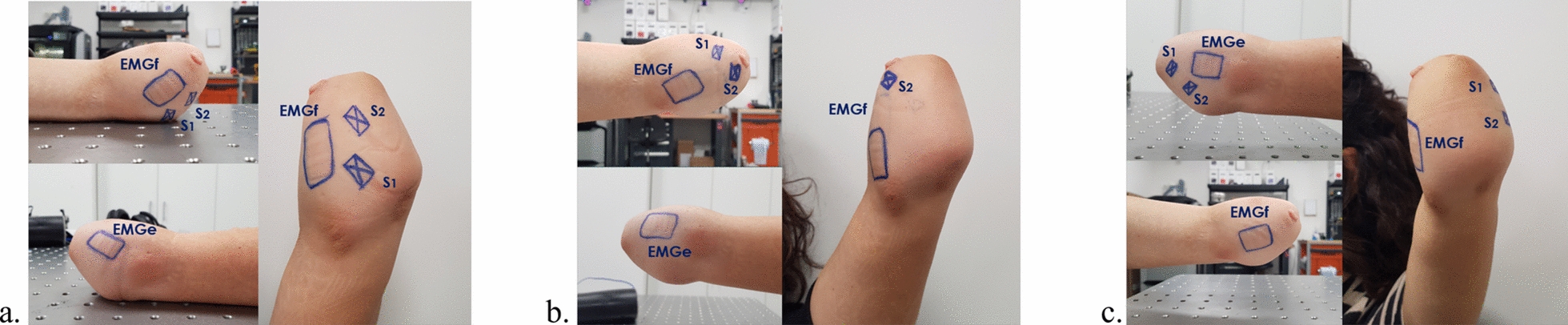
Different electrode configurations tested for the prosthetic user. (**a**) Electrode placement based on the general anatomy of the forearm, (**b**) Placement aimed at stimulating the finger flexor muscle guided by the participant’s feedback, and (**c**) Electrodes positioned over the finger extensor muscles, again adjusted according to the participant’s feedback. Legend: EMGf=EMG electrode on the wrist flexor muscle, EMGe=EMG electrode on the wrist extensor muscle, S1, S2=stimulation electrodes.

For the able-bodied participants, the electrode placement considerations were the same as those described in the previous section. As for the prosthetic user an initial experiment aimed to identify the optimal position for the stimulation electrodes on the residual finger flexor muscle in the stump. Several configurations were tested, ultimately resulting in the selection of three configurations, as depicted in Fig. 5. In Fig. 5a, the electrodes were initially positioned based on the general anatomy of the forearm. In Fig. 5b, the electrodes were adjusted to more accurately target the finger flexor muscles, guided by the participant’s feedback to ensure effective stimulation. Finally, in Fig. 5c, the electrodes were placed over the finger extensor muscles, again adjusted according to the participant’s feedback to optimize the stimulation.

#### Marker placement and hand posture analysis

For both the able-bodied participants and the prosthetic user, the considerations and methodologies employed for marker placement and hand posture analysis were consistent with those utilized in previous sections.

#### Experimental procedure

This experimental session was divided into two distinct parts. The first part focused on defining specific stimulation signals that correspond to minimal and maximal finger extension for each participant. In the second part, these signals were employed to conduct the Cylinder Discrimination test, where participants were required to identify the size of two different cylinders based on the sensory feedback received from the electrodes.

**Fig. 6:**
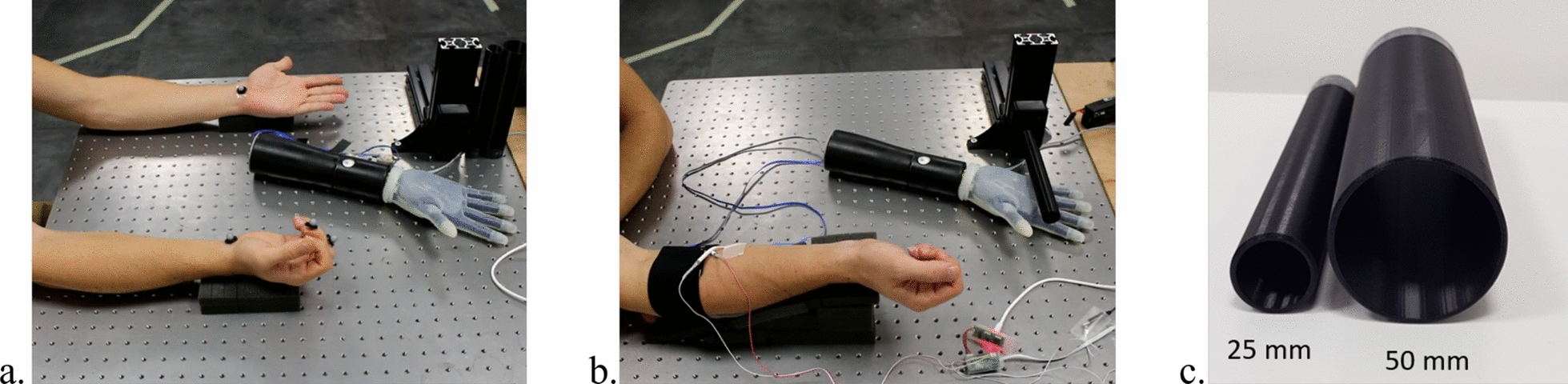
Representation of different parts of the second experimental session depicting (**a**) the participant performing signal calibration to define specific stimulation signals corresponding to maximal and minimal finger extension per participant, (**b**) cylinder discrimination, and (**c**) the dimensions of the two cylinders utilized during the cylinder discrimination part.

1. **Signal Definition**
  The objective of this experiment was to determine the stimulation signals that induced minimal and maximal perceived finger extension. To establish a baseline measurement, participants were instructed to use their left hand to manually replicate the position they perceived in their stationary right hand, which remained at rest (see Fig. 6a). This allowed us to quantify the initial $\hbox {D}_{tot}$ value, serving as a reference for subsequent measurements. After recording the baseline, participants underwent stimulation trials with five different frequencies: 30Hz, 100Hz, 300Hz, 1000Hz, and 3000Hz. For each frequency, a range of voltage amplitudes was applied. The tested amplitudes spanned from the sensory threshold *Vth* to the discomfort threshold *Vmax*, both determined in the previous sections. During stimulation, participants were again asked to replicate the position they perceived in their right hand using their left hand. The corresponding $\hbox {D}_{tot}$ values were recorded for each tested frequency-amplitude combination. The objective was to measure the $\hbox {D}_{tot}$ while different stimulation parameters were applied, determining which combinations resulted in the perception of the greatest and smallest finger extensions compared to the baseline. These stimulation settings were then selected as the signals corresponding to maximal and minimal perceived finger extensions, respectively. For the prosthetic user, the same procedure was conducted, but the analysis was performed separately for the three electrode configurations previously defined.
2. **Cylinder Discrimination**
  Following the identification of the stimulation signals corresponding to minimal and maximal finger extension, the next phase of the experiment involved the Cylinder Discrimination task (Fig. 6b). The aim of this task was to determine if participants could use the previously established stimulation signals to distinguish between objects of different sizes based on proprioceptive feedback.
  Participants were seated comfortably and blindfolded to prevent visual cues from influencing their perception. They wore noise-canceling headphones playing white noise throughout the experiment to eliminate auditory cues. This was combined with blindfolding and randomization of object presentation to reduce the influence of incidental feedback. Surface EMG electrodes were placed on the wrist flexor and extensor muscles, which allowed participants to control the SoftHand Pro prosthetic hand through muscle activity. The participants were then instructed to grasp two 3D-printed cylinders with diameters of 25 mm and 50 mm, depicted in Fig. 6c. The grasping was performed for a fixed duration. The encoder of the prosthetic hand provided a proportional measure of the grasp degree, which was transmitted to a controller. The controller then sent back the corresponding stimulation signal based on the previously identified parameters. Using these feedback signals, participants were required to determine which cylinder they had grasped based on their perceived hand position, indicating which one they believed was larger.
  Each participant completed a total of 25 trials, with a 10-second rest interval between each trial. The order of cylinder presentation was randomized to ensure there was no bias, with either the larger or smaller cylinder presented first. It is important to note that standard psychophysical forced-choice procedures were not employed in this experiment, which could be seen as a limitation of the study.
  During the experiments, the muscles targeted for stimulation needed to be relaxed, an approach consistent with prior TES studies that required muscle relaxation to maximize percept clarity [20]. Therefore, the EMG-controlled grasping and subsequent stimulation were conducted in separate phases to maintain muscle relaxation. Specifically, participants were instructed to close the prosthetic hand using myoelectric control for a fixed duration of 4 seconds. This time window was sufficient to complete the grasp regardless of the object presented and was deliberately kept constant to prevent participants from inferring object size based on the duration or effort of their own muscle contraction. After this interval, participants were asked to fully relax their muscles. Only once muscle relaxation was confirmed, the TES was delivered to the flexor digitorum profundus muscle. This separation ensured that the muscle spindles were effectively stimulated without interference from muscle contractions involved in controlling the prosthetic hand. By adopting this approach, we aimed to isolate the effects of electrical stimulation from sEMG recordings.
  The parameters utilized to assess the performances include Precision, Sensitivity, Specificity, Accuracy, F-score, and Area Under the Curve (AUC). In our analysis, Precision measures the system’s ability to minimize false positives, Sensitivity detects true positives, and Specificity minimizes false negatives. Accuracy provides an overall correctness measure. The F-score balances precision and sensitivity. AUC assesses the classifier’s ability to distinguish between classes across thresholds, with higher values indicating better discrimination. These metrics were calculated using MATLAB’s built-in functions and libraries. Specifically, the |confusionmat| function was employed to generate the confusion matrix from the actual and predicted labels. From this matrix, precision, sensitivity, specificity, and accuracy were derived by extracting true positives, false positives, true negatives, and false negatives. The F-score was calculated as the harmonic mean of precision and sensitivity. The AUC was determined using the |perfcurve| function, which computes the Receiver Operating Characteristic (ROC) curve and its area.

## Results

### TES affects hand proprioception

#### Calibration procedure for optimal stimulation parameters

**Fig. 7:**
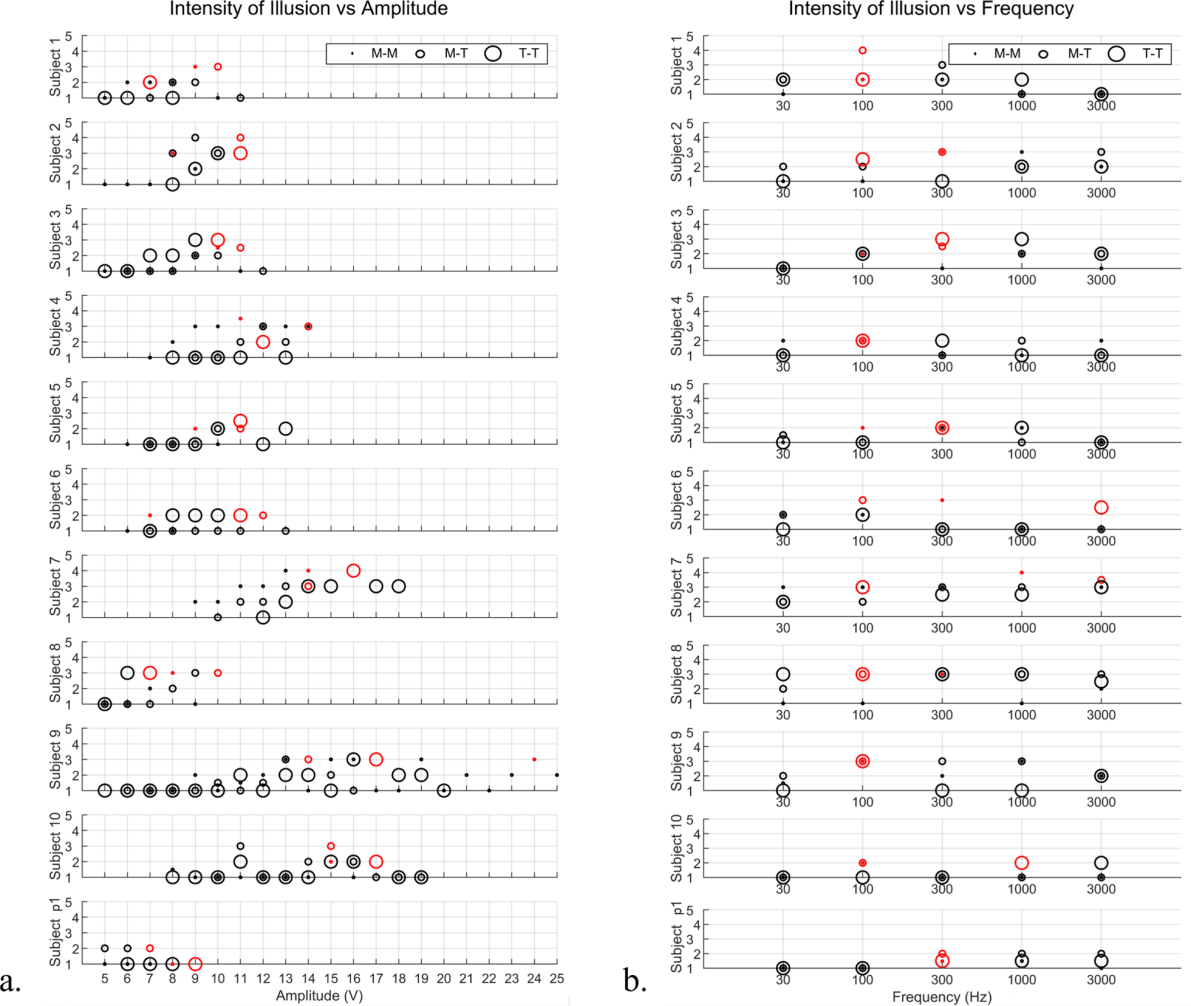
Scores assigned by the ten participants (Subject 1 to 10) and the prosthetic user (Subject p1) to quantify the illusory intensity of the (**a**) amplitudes and (**b**) five frequencies (30 Hz, 100 Hz, 300 Hz, 1000 Hz, 3000 Hz) tested with electrodes in M-M, M-T, and T-T. The scores of the chosen optimal amplitudes are highlighted in red. [1–nothing, 2–barely perceivable, 3–perceivable, 4–strong, 5–very strong]

The primary objective of the calibration procedure was to determine the appropriate voltage and frequency for the stimulation signals. Participants assigned scores to various combinations of voltage and frequency, and the results are depicted in Fig. 7a for amplitude values and Fig. 7b for frequency values. Based on these scores, for each of the three electrode positioning configurations (M-M, M-T, T-T), we identified the optimal voltage and frequency values for each participant highlighted in red, representing the stimulation parameters that elicited the strongest kinesthetic illusion.

#### Hand-matching task

**Fig. 8:**
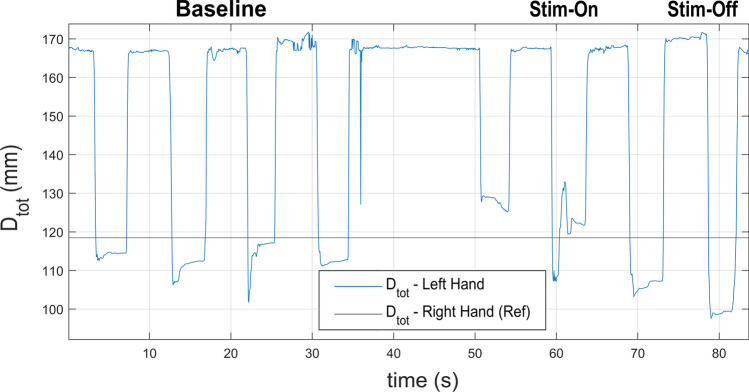
Temporal variation of $\hbox {D}_{tot}$ during the Hand-Matching task, derived from the mean of three distances: $\hbox {D}_1$, $\hbox {D}_2$, and D3. This figure illustrates the temporal trend of D*tot* recorded during the experiment for one participant in the Hand-Matching task. The task was performed four times to establish the baseline position, followed by two sessions with electrode stimulation (Stim-On), and then two sessions post-stimulation (Stim-Off)

After obtaining the optimal amplitude and frequency values, the Hand-Matching task was performed. Fig. 8 shows an example of the temporal variation in the $\hbox {D}_{tot}$ of a participant’s left hand recorded during a 45$^{\circ }$ session.

**Fig. 9:**
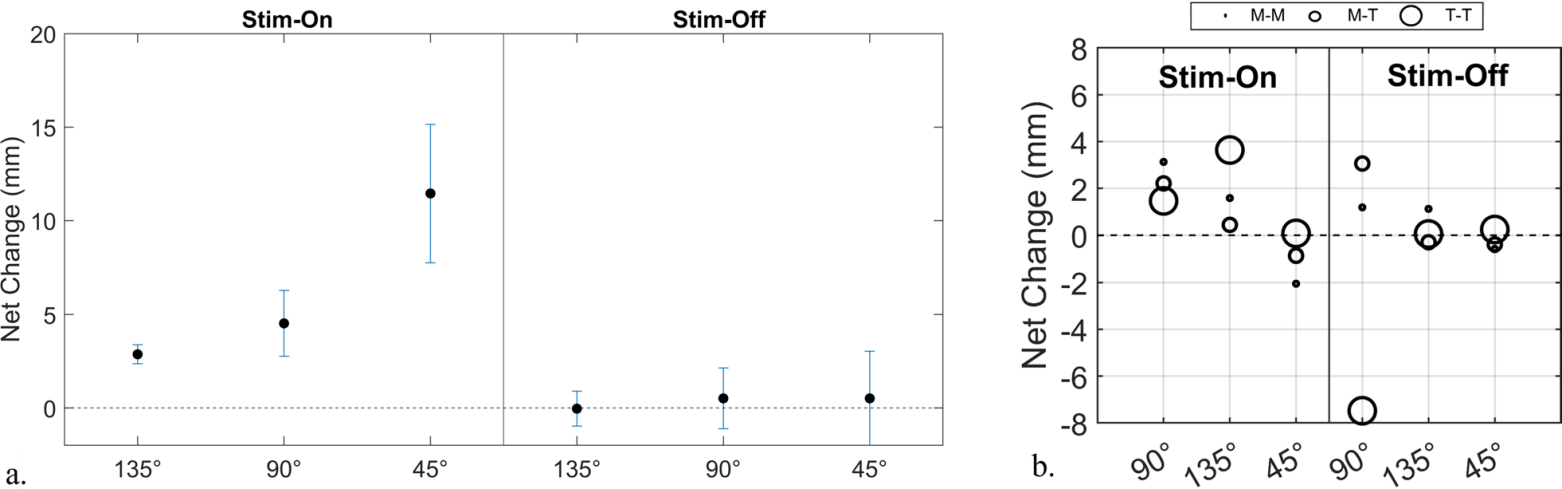
Net change in D_tot_ values between the average left-hand measurements recorded during the stimulation phase and the post-stimulation phase, relative to the baseline. Panel (**a**) shows data from ten able-bodied participants, while panel (**b**) represents the prosthetic user. The net change is defined as the difference between the $\hbox {D}_{tot}$ recorded during (or after) stimulation and the $\hbox {D}_{tot}$ calculated during the Baseline

The net change was analyzed across the three hand positions (45$^{\circ }$, 90$^{\circ }$, and 135$^{\circ }$) for both able-bodied participants and the prosthetic user. The results are summarized in Fig. 9. In particular, Fig. 9a shows the net change calculated during the tests performed by able-bodied participants, distinguishing the three sessions based on the hand’s closure angles: during the 135$^{\circ }$ phase is 2.87 mm ± 0.51, while values of 4.52 mm ± 1.76 and 11.45 mm ± 3.69 are obtained for the 90$^{\circ }$ and 45$^{\circ }$ phases, respectively.

The ten able-bodied participants manifested a maximum net change exceeding 1 centimeter, contrasting with the prosthetic user who registered a maximum net change of less than 4 mm, as shown in Fig. 9b.

#### Data analysis

The statistical analysis revealed significant differences in hand-matching performance during stimulation, confirming that TES modulated participants’ perceived hand posture.

A Kolmogorov-Smirnov test was performed to assess data distribution. Since the data did not follow a normal distribution (p < 0.05), we used non-parametric tests. The Wilcoxon-Mann-Whitney test indicated that the Stim-On phase significantly differed from the Baseline in all three tested angles (p < 0.05): in particular, we obtained p-values of 0.002 (for the 45$^{\circ }$ session), 0.006 (for the 90$^{\circ }$ session), and 0.002 (for the 135$^{\circ }$ session).

In contrast, statistical tests comparing the Baseline and Stim-Off phases showed no significant differences, suggesting that the induced effects did not persist once stimulation ceased. Specifically, we obtained p-values of 0.32 (for the 45$^{\circ }$ session), 0.63 (for the 90$^{\circ }$ session), and 0.92 (for the 135$^{\circ }$ session).

### TES displays prosthesis status

#### Signal definition

**Fig. 10:**
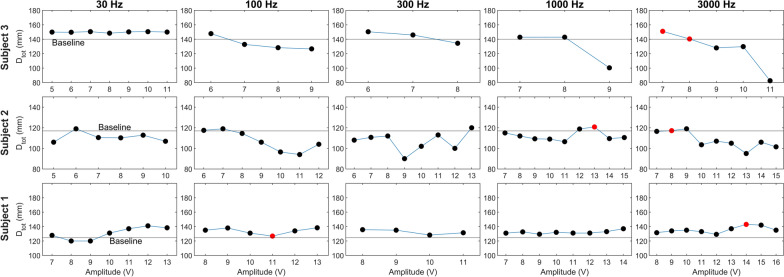
Results of the signal calibration experiment conducted on able-bodied participants (Participant 1, Participant 2, and Participant 3). The red markers indicate the amplitude-frequency pairs selected to generate the maximum and minimum kinesthetic perceptions

Baseline $\hbox {D}_{tot}$ values, measured before stimulation, differed across participants. For the three able-bodied participants, these baseline values were 139.9 mm, 117.0 mm, and 124.7 mm, respectively. During the stimulation trials, we recorded $\hbox {D}_{tot}$ values for each tested frequency-amplitude combination. The resulting values, depicted in Fig. 10, allowed us to identify the parameter pairs that elicited the lowest and highest deviations from the Baseline for each participant. From these measurements, we determined the specific voltage and frequency parameters associated with the minimal and maximal perceived finger extensions for each participant.

**Fig. 11:**
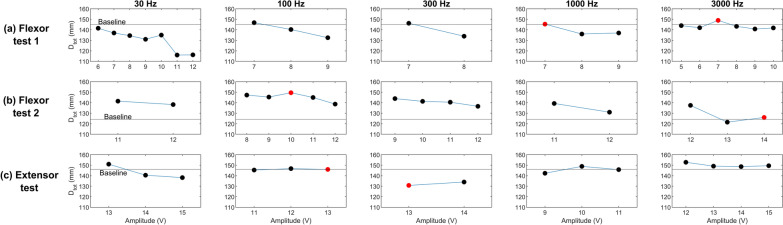
Results of the signal calibration experiment conducted on the prosthetic user, with electrodes positioned according to three different conditions: (**a**) based on the anatomy of the residual limb, (**b**) based on the finger flexor muscle location guided by the participant’s feedback, and (**c**) based on the finger extensor muscles. The red markers indicate the values corresponding to the selected amplitude-frequency pairs that generated the maximum and minimum kinesthetic perceptions

For the prosthetic user, results varied depending on the electrode placement. The baseline measurements for the three cases were 145 mm, 124.13 mm, and 146.1 mm, respectively. Using the same methodology applied to able-bodied participants, we identified the frequency-amplitude pairs that elicited the minimal and maximal perceived finger extensions for each configuration. The results are summarized in Fig. 11.

#### Cylinder discrimination

After identifying the signals associated with the sensations of maximum and minimum extension for each participant, the subsequent step involved the Cylinder Discrimination experiment. A confusion matrix was constructed to evaluate the results of each participant comprehensively. Tables 1 and 2 present results for able-bodied participants and the prosthetic user, respectively. As depicted in Table 1, Participant 1 demonstrated the highest discrimination Accuracy at 92%. Participant 3 also exhibited notable Accuracy in identifying the larger object, achieving 80%, while Participant 2 showed a discrimination Accuracy of 68%. The results of the Cylinder Discrimination performed by the prosthetic user are shown in Table 2. Notably, in the second and third configurations, the Accuracy in discriminating the correct cylinder is very high (100% and 92%, respectively), contrasting with the first case where the Accuracy is 64%.

**Table 1:** Results of Cylinder Discrimination in Preliminary Studies on Able-Bodied Participants

	Participant 1	Participant 2	Participant 3
Precision	0.91	0.67	0.77
Sensitivity	0.91	0.67	0.83
Specificity	0.92	0.69	0.77
Accuracy	0.92	0.68	0.80
F-score	0.91	0.67	0.80
AUC	0.92	0.68	0.80

**Table 2:** Performance of Prosthetic Users in the Cylinder Discrimination Experiment

	Flexor Test 1	Flexor Test 2	Extensor Test
Precision	0.71	1.00	0.88
Sensitivity	0.67	1.00	1.00
Specificity	0.60	1.00	0.82
Accuracy	0.64	1.00	0.92
F-score	0.69	1.00	0.93
AUC	0.63	1.00	0.94

## Discussion

### TES affects hand proprioception

In the first experimental session, able-bodied participants consistently reported a distinct perception of finger extension during Transcutaneous Electrical Stimulation (TES). This finding supports the idea that an intact sensory-motor system facilitates effective integration of artificial input, enabling the emergence of movement-related perceptions. While the prosthetic user exhibited a less pronounced response, a clear sensation of finger extension was still reported across multiple trials. These results suggest that TES can elicit structured and perceivable feedback even in the absence of natural proprioception, reinforcing the feasibility of its use in prosthetic applications.

Importantly, participants were generally able to distinguish the intended illusion of finger movement from confounding tactile or tingling sensations at the stimulation site. In some instances, variations in perceived intensity across stimuli were reported, indicating that tactile cues may have partially influenced task performance. Nevertheless, these did not appear to fully obscure the perception of movement, underscoring the potential of TES to induce functionally relevant perceptual effects. This highlights the importance of refining stimulation parameters–such as electrode positioning and waveform shaping–to enhance selectivity and minimize unintended tactile interference.

The Hand-Matching task further corroborated the perceptual findings. The strongest stimulation effects were observed at 45$^{\circ }$, where the greater range of motion likely enhanced the detectability of hand position changes. Conversely, the effect was attenuated at 135$^{\circ }$, possibly due to biomechanical constraints limiting finger extension in that posture. These findings align with Weber’s Law [27], which suggests that the discriminability of a stimulus depends on its baseline intensity. Notably, the stimulation-induced displacement remained significant across all tested angles, suggesting robustness of the perceptual effect.

During the Stim-Off phase, able-bodied participants showed a return to baseline performance, with near-zero net changes in hand matching. This indicates that short-term TES does not produce lasting residual effects and that the perceptual modulation is reversible and temporally bounded. Although promising for dynamic feedback applications, this finding also suggests that repeated or prolonged exposure might be needed to explore potential adaptation or training effects–a direction worth pursuing in future longitudinal studies.

Regarding the prosthetic user, the stimulation produced more modest perceptual responses. While finger extension sensations were reported, the magnitude of hand-matching deviations remained less pronounced across all tested conditions. This attenuated sensitivity may reflect individual neurophysiological factors, such as cortical reorganization or long-term sensory deprivation. Notably, the participant in question was congenitally limb-absent, a condition that differs fundamentally from post-amputation profiles in terms of somatosensory representation and neural plasticity. Such differences may critically shape the efficacy of TES-based interventions and underscore the need to account for user-specific factors when designing feedback protocols.

Although limited to a single case, the prosthetic user’s ability to perceive directionally appropriate effects suggests that the technique holds promise even in cases of congenital limb difference. Future studies should therefore aim to include broader participant cohorts–both post-amputation and congenital–to explore variability in responsiveness and to statistically characterize the method’s efficacy across populations.

### TES displays prosthesis status

The Signal Definition experiment enabled the identification of stimulation parameters capable of eliciting consistent perceptions of minimal and maximal finger extension in each participant. Among able-bodied individuals, sensitivity to TES varied, likely influenced by physiological and psychological factors. Muscular micro-contractions–previously documented in the literature [11, 28]–may have contributed to differences in perceptual thresholds. In addition, individual psychological states such as stress or anxiety, known to affect proprioceptive acuity [29], could have played a role in modulating responses. These factors highlight the importance of personalized calibration and call for further investigation into the neuropsychological variables that influence artificial sensory feedback.

In the case of the prosthetic user, the signal calibration experiment revealed notable variability in perceived hand position across trials, suggesting the absence of a stable internal model of the missing limb. The participant consistently reported the perceived resting state of the prosthetic hand as relatively open, yet this perception lacked consistency over time. This variability may stem from congenital limb absence, which is associated with distinct cortical organization and altered body representations compared to acquired amputations. These findings emphasize the need to consider individual neurodevelopmental backgrounds when designing and evaluating sensory feedback strategies for prosthetic users.

During the discrimination task, performance in the prosthetic user was highly dependent on electrode positioning. Accurate perceptual responses were observed only when electrodes were positioned beyond standard anatomical configurations, underscoring the necessity of user-specific stimulation strategies. Despite a generally reduced response compared to able-bodied participants, the prosthetic user demonstrated consistent and repeatable effects, confirming that TES can still evoke meaningful perceptual responses even in the absence of natural proprioceptive pathways. These promising outcomes reinforce the feasibility of TES for modulating sensorimotor experience in prosthetic applications.

From a technical perspective, TES is inherently limited by its superficial action, which may lead to co-activation of nearby muscles. Although the flexor digitorum profundus (FDP) was targeted, it is plausible that adjacent muscles such as the flexor digitorum superficialis (FDS) were also recruited, potentially influencing the perceptual outcome. This limitation, inherent to non-invasive stimulation methods, was carefully considered in data interpretation. Future work may address this constraint through refined electrode targeting, muscle-specific activation strategies, or hybrid feedback techniques.

Encouragingly, this pilot study provides promising early evidence that transcutaneous electrical stimulation (TES) can modulate proprioceptive sensations in both able-bodied individuals and, importantly, in a prosthetic user. Due to hardware constraints at the time of the study, the electrodes were not yet integrated into the prosthetic socket, which prevented the execution of functional user-in-the-loop tasks. Instead, a controlled discrimination paradigm was adopted to isolate and evaluate the perceptual effects of stimulation. We acknowledge that the current findings should not be interpreted as evidence of functional prosthetic success. Rather, this study serves as an initial step toward evaluating whether TES-induced proprioceptive illusions elicited via TES could be used in a closed-loop prosthetic control scenario. While the inclusion of a single prosthetic participant reflects the exploratory scope of the work, the study was designed to assess the feasibility of perceptual induction through TES and to establish a foundation for future experimental development. Despite the limited sample size, the protocol consistently elicited clear and repeatable perceptual responses across participants. In the prosthetic user, although the effects were less pronounced than those observed in able-bodied individuals, they were nonetheless structured and reproducible–demonstrating that meaningful sensorimotor feedback can still emerge in the absence of natural proprioceptive input. These findings reinforce the feasibility of TES as a non-invasive approach to enhancing artificial somatosensory feedback and underscore the critical role of factors such as electrode placement and individual responsiveness in shaping the effectiveness of the intervention.

Our findings should also be interpreted in light of recent studies that have extended electrotactile proprioceptive feedback to other joints. Han et al. [21] demonstrated that proprioceptive information for the wrist can be conveyed through spatially coded cutaneous stimulation using multichannel arrays. Ravichandran et al. [22] showed that frequency-modulated fingertip stimulation improves finger-aperture tracking performance and supports motor learning. Both studies highlight the feasibility of electrotactile substitution, but they operate at a cutaneous level and require structured training paradigms. By contrast, our work employs single-channel TES targeted to the flexor digitorum profundus, eliciting proprioceptive illusions of muscle stretch rather than cutaneous cues. This muscle-based approach provides a different encoding strategy, in which the stimulation intensity is directly proportional to prosthetic aperture.

In contrast to these structured training paradigms, our study adopted a simplified pilot approach, focusing on whether TES-induced proprioceptive illusions could be integrated into a prosthetic control loop, even in the absence of extensive training. Although our discrimination accuracy was moderate compared to the high performances reported by Han and colleagues, this discrepancy likely reflects the absence of extensive training in our pilot protocol and the more direct integration with myoelectric prosthesis control. Thus, our study complements previous work by showing that TES-evoked proprioceptive illusions can be exploited in a prosthetic context, paving the way toward future paradigms that combine the immediacy of muscle-based illusions with the robustness of cutaneous substitution schemes.

Looking ahead, several avenues can enhance the performance and acceptance of TES in prosthetic contexts. One possibility is to combine TES with complementary modalities, such as skin-stretch or vibrotactile feedback, to enrich the perceptual experience. Additionally, adapting the protocol to joints with simpler biomechanics (e.g., the knee) could improve response specificity. An exciting prospect involves the development of a non-invasive analogue to the Agonist-Antagonist Myoneural Interface (AMI), which restores bidirectional proprioceptive loops via surgically connected muscle grafts [30, 31]. Non-invasive alternatives could simulate similar feedback by coupling electromyographic monitoring of the agonist muscle with real-time TES of the antagonist.

Despite certain limitations, TES remains a highly attractive modality due to its non-invasive nature, ease of implementation, and adaptability across users. These features position it as a promising solution for enhancing proprioceptive awareness in prosthetic systems. However, challenges remain. TES sensations may differ from natural proprioceptive feedback and, in some cases, may be perceived as uncomfortable or unintuitive [32]. Additionally, some users may prefer single-modality feedback over multimodal integration [33], suggesting the need for careful system design tailored to user preferences.

In conclusion, the results of this study demonstrate the feasibility and potential of TES for providing structured proprioceptive information in prosthetic users. Expanding the participant cohort, especially among prosthetic users with diverse backgrounds, will be crucial for validating and generalizing these findings to broader clinical applications. By overcoming these limitations, we can enhance the effectiveness, user experience, and long-term viability of TES as a feedback tool for prosthetic control.

## Conclusion

In this study, we aimed to establish and validate a novel protocol for applying TES to a distinct muscular group, specifically targeting the flexor digitorum profundus muscle. Additionally, we investigated the TES for proprioceptive feedback in order to improve the accuracy of finger position perception in a prosthetic user. This pioneering investigation represents a pilot study, marking the first application of TES in this context.

To achieve this, two experiments were conducted. Initially, the stimulation was applied to the flexor digitorum profundus muscle to induce a perception of finger extension. Measurements taken from ten able-bodied participants before and during the application of the electrical stimulus confirmed the occurrence of a statistically significant perception of finger extension. This demonstrated the technique’s potential to effectively modulate proprioceptive perceptions, even in complex muscles like the flexor digitorum profundus muscle.

In the second part of our study, we shifted our focus to implementing TES for proprioceptive feedback, including the usage of a prosthetic device in our tests. Tests conducted on able-bodied participants were promising, showing high accuracy. Conversely, tests on a specific prosthesis user revealed low sensitivity to the technique. Nonetheless, when the experiment was repeated with different electrode positions, almost perfect accuracy was achieved, indicating the potential of electrical stimulation to enhance sensory perception in prosthetic users.

In summary, this study establishes a robust foundation for future investigations aimed at improving sensory feedback and motor control in individuals with limb loss through electrical stimulation. The findings in this study highlight the potential for this technique to significantly enhance the quality of life for prosthetic users, paving the way for advancements in neurorehabilitation and assistive technology. The non-invasive, economical nature of this technique, coupled with its ability to induce substantial proprioceptive sensations, makes it a valuable tool for future developments in this field.

## Data Availability

Data is provided within the manuscript.
